# Metabolomics dataset of zebrafish optic nerve regeneration after injury

**DOI:** 10.1016/j.dib.2023.109102

**Published:** 2023-04-18

**Authors:** Sean D. Meehan, Mengming Hu, Matthew B. Veldman, Sanjoy K. Bhattacharya

**Affiliations:** a Bascom Palmer Eye Institute University of Miami, 1638 NW 10th Avenue, Suite 707A, Miami, FL, 33136, United States; b Miami Integrative Metabolomics Research Center, Miami, FL, 33136, United States; c University of Miami Miller School of Medicine, Miami, FL, 33136, United States; d Department of Cell Biology, Neurobiology, and Anatomy, Medical College of Wisconsin, Milwaukee, WI, 53226, United States; e Department of Ophthalmology and Visual Sciences, Medical College of Wisconsin, Milwaukee, WI, 53226, United States

**Keywords:** Zebrafish, Optic nerve, Regeneration, Metabolomics, High-performance liquid chromatography

## Abstract

Zebrafish (*Danio rerio*) have the capacity for successful adult optic nerve regeneration. In contrast, mammals lack this intrinsic ability and undergo irreversible neurodegeneration seen in glaucoma and other optic neuropathies. Optic nerve regeneration is often studied using optic nerve crush, a mechanical neurodegenerative model. Untargeted metabolomic studies within successful regenerative models are deficient. Evaluation of tissue metabolomic changes in active zebrafish optic nerve regeneration can elucidate prioritized metabolite pathways that can be targeted in mammalian systems for therapeutic development. Female and male (6 month to 1 year old wild type) right zebrafish optic nerves were crushed and collected three days after. Contralateral, uninjured optic nerves were collected as controls. The tissue was dissected from euthanized fish and frozen on dry ice. Samples were pooled for each category (female crush, female control, male crush, male control) and pooled at *n* = 31 to obtain sufficient metabolite concentrations for analysis. Optic nerve regeneration at 3 days post crush was demonstrated by microscope visualization of GFP fluorescence in *Tg(gap43:GFP)* transgenic fish. Metabolites were extracted using a Precellys Homogenizer and a serial extraction method: (1) 1:1 Methanol/Water and (2) 8:1:1 Acetonitrile/Methanol/Acetone. Metabolites were analyzed by untargeted liquid chromatography-mass spectrometry (LC MS-MS) profiling using a Q-Exactive Orbitrap instrument coupled with Vanquish Horizon Binary UHPLC LC-MS system. Metabolites were identified and quantified using Compound Discoverer 3.3 and isotopic internal metabolites standards.

**Specifications Table**

**Table:** 

Subject	Ophthalmology
Specific subject area	Zebrafish Metabolites in neuronal regeneration
Type of data	Chart Chromatogram Figure Graph Image Spectra
How the data were acquired	High-Performance Liquid Chromatography, Q Exactive Orbitrap Mass Spectrometer
Data format	Raw Analyzed Filtered
Description of data collection	Optic nerves were collected from Zebrafish (*Danio rerio)* 3 days post crush injury. Approximately 31 optic nerves each were pooled for Female and Male Samples. Untargeted metabolomics was performed and analyzed using high-performance liquid chromatography and mass spectrometry. Internal standards were used to normalize data.
Data source location	Bascom Palmer Eye Institute, Miller School of Medicine, University of Miami, Miami, FL 33,136, USA
Data accessibility	This study is available at the NIH Common Fund's National Metabolomics Data Repository (NMDR) website, the Metabolomics Workbench, https://www.metabolomicsworkbench.org where it has been assigned Study ID **ST002444**. The data can be accessed directly via its Project DOI: **doi: 10.21228/M83 × 51** This work is supported by NIH grant **U01EY027257, P30EY014801**.

## Values of the Data

• This data provides valuable insight on the metabolic shifts during the Zebrafish's intrinsic regenerative abilities in optic nerve regeneration after injury
• This data can provide information of natural endogenous metabolites in Zebrafish that are associated with axonal regeneration.
• This data can be used for marker discovery to guide future targeted metabolite analyses and potential therapeutic development

## 1 Objective

This dataset's goal is to provide foundational metabolomic data on the intrinsically regenerative organism, *Danio rerio* (zebrafish). After optic nerve crush/injury, zebrafish can regrow their optic nerve without therapeutic intervention. This dataset contains optic nerve metabolite information at 3 days post crush approximately when the optic nerve has regenerated up to the optic chiasm. We hope this dataset can be used to identify prioritized metabolomics pathways in regeneration and compared with the nonregenerative mammalian ocular systems.

## 2 Data Description

**Fig. 1:**
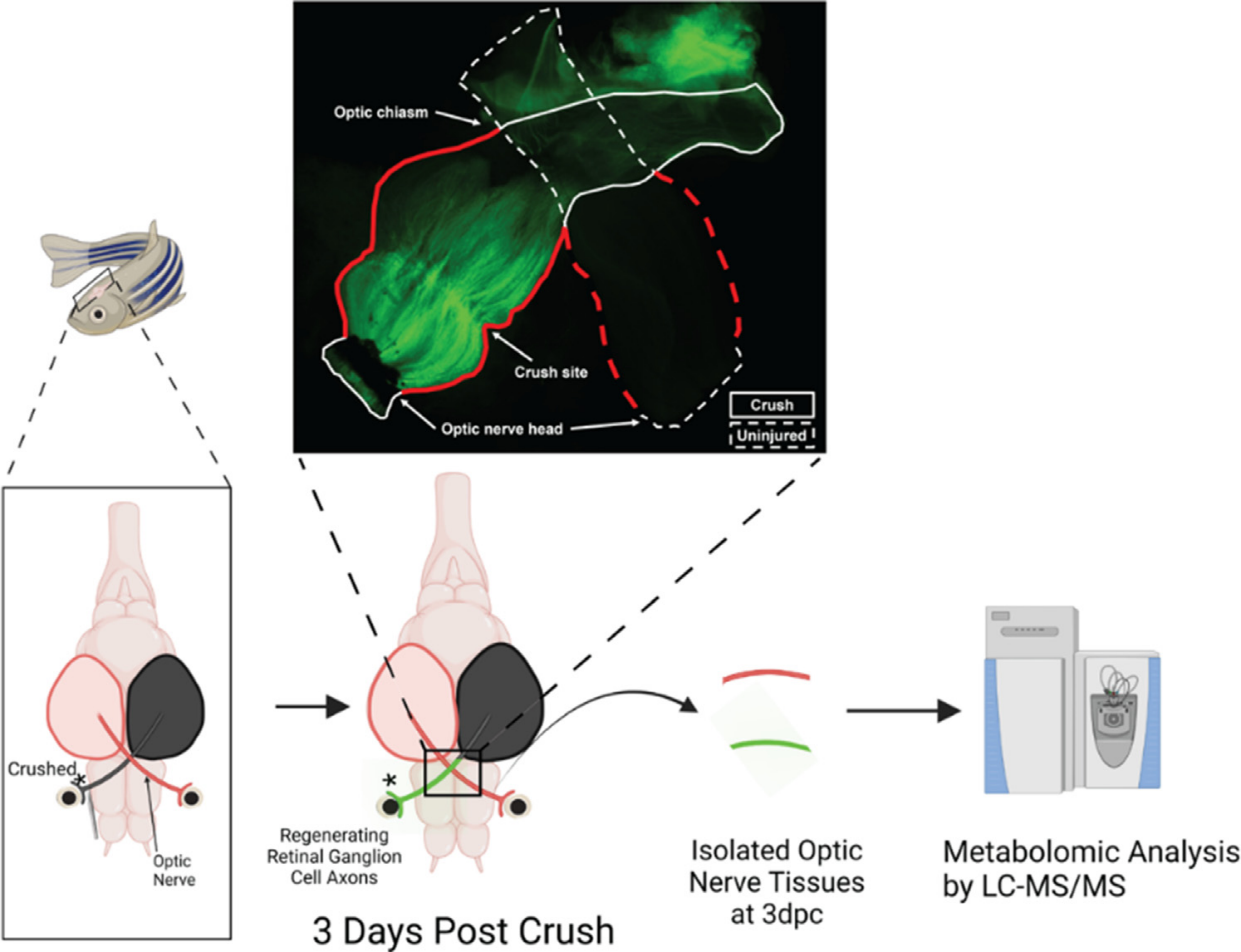
Strategy for tissue harvesting from regenerating and control uninjured zebrafish optic nerves. For illustration purposes *Tg(gap43:GFP)* were used to demonstrate the length of regenerating GFP+ axons, solid outline, in the 3 day post crush optic nerve and lack of regenerating axons in the control nerve, dotted outline. The collected pre-chiasm tissue from each optic nerve is highlighted in red outline in the upper panel. In the lower panels a cartoon describes the anatomical locations of the injury site and the tissues collected. The crushed optic nerve is black and then turns green during regeneration and the uninjured control nerve is in red.

Zebrafish (*Danio rerio*) have the ability to successfully regenerate damaged retina and optic nerves without therapeutic intervention [1]. Humans lack this innate regenerative ability. Some metabolites have been discovered that support mammalian nerve regeneration [2,3]. However, a full metabolomic profile of the regenerating zebrafish optic nerve has not been established. This profile may increase the list of possible metabolites that stimulate regenerative pathways. Optic nerve regeneration is often studied in organisms using an optic nerve crush degenerative mode that involves forceps crushing the optic nerve for 5 s. The regenerating optic nerve is visualized using a transgenic *Tg(gap43:GFP) zebrafish. GAP-43 is a known neuronal growth marker in both zebrafish and mammals.* Here, we present a metabolomic dataset of optic nerve metabolites for control and crush optic nerves 3 days post crush from zebrafish males and females. Approximately 30 optic nerves were pooled per group. Pooling tissue samples is necessary as metabolite signals are relatively weak and zebrafish tissues are small. At 3 days post crush, regenerated axons have reached the optic chiasm (Fig. 1). Metabolites were extracted using a serial extraction method (1: Methanol/Water; 2: Acetonitrile/Methanol/Acetone) combined with a Precellys Touch Homogenizer. After extraction and sample drying, pooled quality control samples were created for batch-to-batch normalization.

**Fig. 2:**
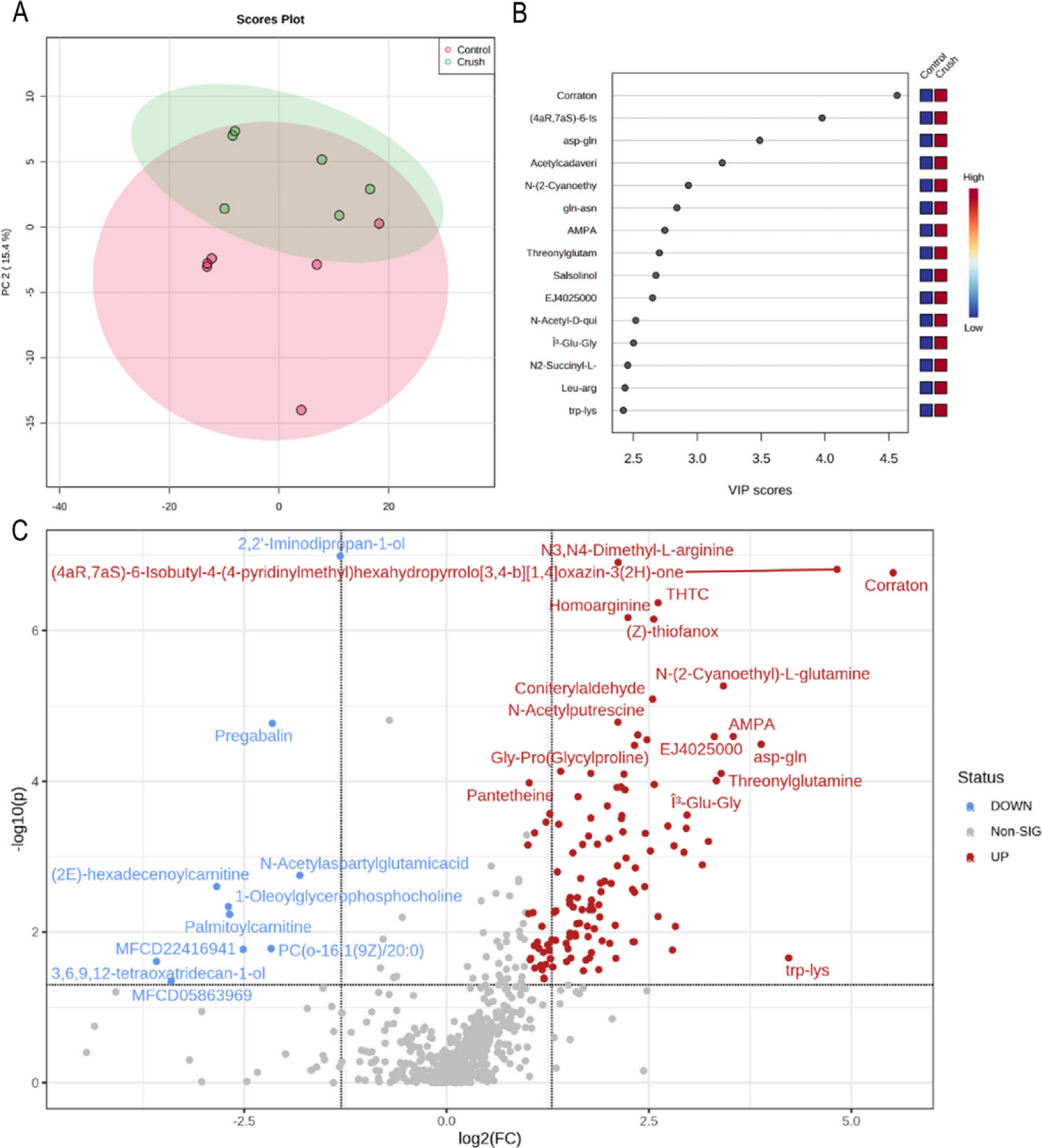
Multivariate analysis of metabolites for zebrafish crush and control optic nerve groups 3 days post crush. (A) Principal component analysis (PCA) 2-D scores plot of metabolite profiling data. (B) Partial Least Squared Discriminant Analysis with associated Variable Importance in Projection (VIP) scores. The colored boxes on the right indicate the relative concentrations of the corresponding metabolite in each group. Red indicates high concentration and blue indicates low concentrations. (C) Volcano plot of significant up and down regulated metabolites (FDR<0.05, FC>2). Labels are provided per metabolites. Red labeling indicates increased Crush/Control fold change while blue indicates decreased Crush/Control fold change. gray dots are non-significant metabolites.

Metabolites were separated using a HILIC column on a Vanquish Ultra High Performance Liquid Chromatograph. Mass spectra were generated using a Q Exactive Orbitrap Mass Spectrometer on positive and negative ion modes with three technical replicates. Mass Spectrometry files were aligned, and metabolites were identified using Compound Discoverer 3.3. Metabolite peak areas were normalized using six isotopic internal standards from different metabolite classes and retention times. Metaboanalyst was used to process the data and generate principal component analysis (PCA), partial least squares discriminant analysis (PLS-DA), volcano, and heatmap plots. T.test and Volcano plot analysis found 86 significantly changed metabolites between control and crush zebrafish optic nerves (Fig. 2). Approximately 90% of the significant metabolites were upregulated in the crush samples.

## 3 Experimental Design, Materials and Methods

### 3.1 Animals

Adult zebrafish, 6 months to 1 year in age, were used for all groups both male and female. The NIH guide for the care and use of laboratory animals was followed and all procedures were approved by the Medical College of Wisconsin IACUC ID AUA1378. For optic nerve crush, animals were deeply anesthetized in 0.033% tricaine methane-sulfonate (MS-222). The right optic nerve was exposed by gently removing the connective tissue on the dorsal half of the eye and rotating the eye ventrally out of the orbit with a number 5 forceps. A nerve crush was then performed using number 5 forceps to crush the nerve ∼0.5–1 mm from the optic nerve head for 5 s. Success was observed by the generation of a translucent stripe in the nerve that completely separated two areas of white myelination with no bleeding. Fish were then revived in fresh aquatic system water in individual tanks. After 1 h the tanks were returned to the fish system and animals were maintained normally with daily feeding until 3 days post injury. For tissue collection, animals were euthanized by overdose of MS-222 and optic nerve removed by dissection from the optic nerve head to the optic chiasm. Female and male zebrafish optic nerves were collected separately as biological samples. Due to the small tissue and metabolomics resolution constraints, optic nerves were pooled to generate higher signal intensities. 31 and 36 crushed optic nerves were pooled for female and male zebrafish samples, respectively. The contralateral uncrushed optic nerves were pooled in the same way. This resulted in 2 female pooled groups: 1 crushed and 1 uncrushed contralateral optic nerves and 2 male pooled groups: 1 crushed and 1 uncrushed contralateral optic nerves.

### 3.2 Optic nerve regeneration visualization

To demonstrate the expected amount of axonal regeneration present in the samples, optic nerve crush was performed on *Tg(gap43:GFP)* transgenic zebrafish[4]. On day 3 post crush injury the animal was euthanized, and the optic nerves connected at the chiasm were dissected out. After fixation in 4% PFA for 1 h at room temperature, the nerves were flat mounted on a slide and imaged for GFP fluorescence.

### 3.3 Metabolite extraction

Tissue samples were stored at −80 °C until ready for extraction. Metabolite extraction from samples was carried out quickly while keeping optic nerve tissues on dry ice to prevent metabolite degradation. Tissues were transferred to 0.5 mL Soft Tissue Lysing Kit Precellys tubes containing beads. Eighty-four microliters of chilled 1:1 MeOH/H2O was added to Precellys tube. Pre-extraction internal standards were added to the tubes: 5 µl of 1 mg/ml Caffeine 13C6, 5 µl of 1 mg/ml d-Glucose 13C6, 5 µl of 1 mg/ml Oleic Acid 13C5, and 1 µl of 5 mg/mL Isoleucine 13C6. Tissues were homogenized using Precellys 24 Touch. Cycle parameters: 2 cycles: 30 s homogenization at 4500 rpm, 10 s of rest. Homogenate was transferred to microcentrifuge tube and centrifuged at 18000xrcf for 20 min at 4 °C. Supernatant was collected and pellet was transferred to Precellys Lysing Kit tube. Add Eighty-four microliters of chilled 8:1:1 Acetonitrile/Methanol/Acetone was added to pellet. The rest of the pre-extraction internal standards were added: 5 µl of 1 mg/ml Caffeine ^13^C_6_, 5 µl of 1 mg/ml d-Glucose ^13^C_6_, 5 µl of 1 mg/ml Oleic Acid ^13^C_5_, 1 µl of 5 mg/mL Isoleucine ^13^C_6_. Final pre-extraction internal standards concentrations are 50 µg/mL. Homogenization cycles were repeated using Precellys 24 Touch. Centrifuge as before and combine second supernatant to first round of collected supernatant. Supernatants were centrifuged at 1800xrcf for 20 min once more to remove any remaining tissue debris. Supernatant was collected and dried by Speedvac. Two extraction blanks were prepared in the same manner as the biological samples.

### 3.4 High performance liquid chromatography and mass spectrometry

Dried samples were reconstituted immediately in 44.75µL of HPLC-MS grade water + 0.1% formic acid. Post-extraction internal standards were added: 25 µl of 5 mg/ml Phenylalanine ^13^C_6_, 2.5 µl of 0.5 mg/ml Uracil ^13^C ^15^N_2_, 1.25 µl of 1 mg/ml Arginine ^13^C_6_, 1.25 µl of 1 mg/ml Serine ^13^C_3_ to each sample_,_ Concentrations of all post-extraction internal standards were 25 µg/mL.. Samples were run as technical triplicates

Pooled quality controls (QCs) containing all compounds representative of a batch were run in separate HPLC-MS vials to account for reproducibility and analyte stability. Pooled QCs were created by taking 10 µL aliquots of each sample and combined into a new vial.

Samples were subjected to fractionation and detection using a Thermo Scientific™ Vanquish™ Horizon Binary UHPLC. An Accucore™ Vanquish™ HILIC Accucore Amide 150 Column (100 mm x 2.1 mm, 1.5 µm, Thermo Scientific) was used to separate compounds with a flow rate of 0.500 mL/min. Positive Mode: Mobile Phase A consisted of 10 mM ammonium formate in 95% acetonitrile with 0.1% formic acid (v/v). Positive Mode: Mobile Phase B consisted of 10 mM ammonium formate 50% acetonitrile with 0.1% formic acid (v/v). Negative Mode: Mobile Phase A consisted of 10 mM ammonium acetate in 95% acetonitrile with 0.1% acetic acid. Negative Mode: 10 mM ammonium acetate in 50% acetonitrile with 0.1% acetic acid. Column temperature was set to 35 °C and injection volume at 5 µL.

The samples were run using a Q Exactive™ mass spectrometer coupled to a heated electrospray ionization (HESI) source. The spray voltage was set to 3.50 kV, capillary temperature to 350 °C, sheath gas to 55, aux gas to 14, sweep gas to 4, and S-Lens RF Level to 30.0. The mass range was set to 67 – 1000 *m/z*, resolution 140,000 for full scan and 35,000 for ddMS^2^. AGC target was set to 1e6 for full scan and 2e5 for ddMS^2^. The max injection time (IT) was 100 s for full scan mode and 50 s for ddMS^2^. The number of microscans was 2, and normalized collision energy (NCE) was set to 20, 35, and 50. Samples were run in both positive and negative ion mode separately. The parameters for negative mode were the same except the spray voltage, which was set to 2.50 kV and capillary temperature to 380 °C.

### 3.5 Metabolite identification and statistical analysis

Metabolites were identified from their Thermo.RAW scans using Compound Discoverer™ 3.3 software. Extraction blanks were used to determine and correct for reagent effects, allow for the creation of exclusions lists, mark background components, and filters the background components from the results table in Compound Discoverer™ 3.3. Pooled QCs were used for initial compound normalization and identification. Metabolite peak areas were normalized by representative internal standard. Peaks with the same annotated metabolite species were merged. Positive and negative ionization modes were aligned and consolidated. The resulting list of metabolites and estimated concentrations were uploaded to MetaboAnalyst. Concentrations were log transformed with no data scaling to achieve normal distribution.

**Fig. 3:**
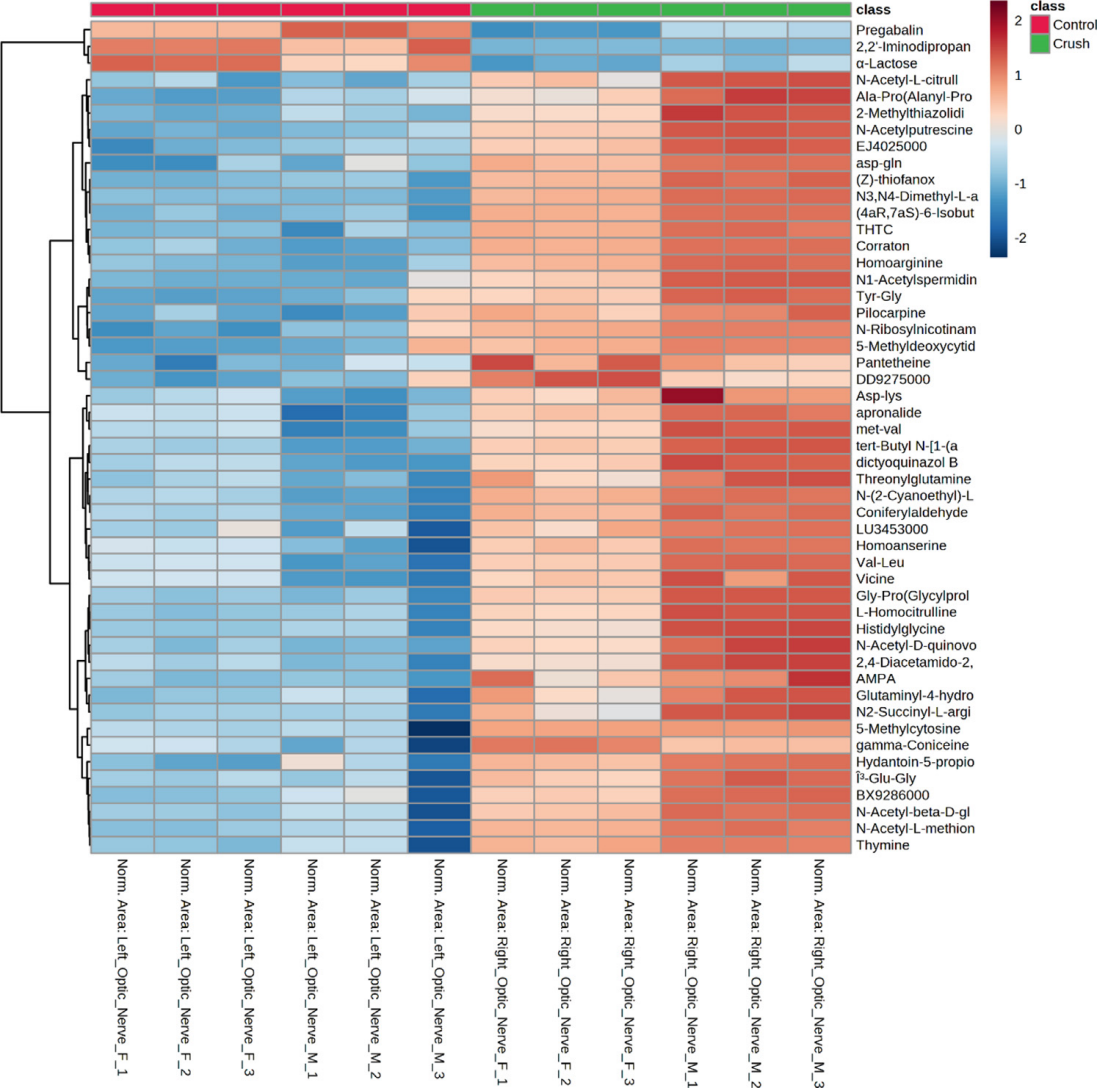
Heatmap of top 50 metabolites for zebrafish crush and control optic nerve groups 3 days post crush. Control (Red) and Crush (Green) groups presented with list of metabolites to the right of heatmap. All left optic nerves were noncrush control while right optic nerves were crush. Male (M) and Female (F) and corresponding technical replicate are indicated in the sample name.

For univariate analysis, a t.test was performed (p-value threshold 0.05)) to identify significant metabolites and generate a volcano plot. A principal component analysis (PCA) 2-D scores plot and partial least square-discriminant analysis (PLS-DA) variable importance in projection (VIP) plots were created. Clustering analysis was performed in the form of a heatmap to show metabolite concentration changes (distance measured using Euclidean, Ward clustering algorithm, standardization using autoscale features) (See Fig. 3).

## Ethics Statements

This study utilized animals (zebrafish, encompassing both genders) only. All animal experiments were performed in compliance with the US National Institutes of Health guide for the care and use of Laboratory animals.

## CRediT authorship contribution statement

**Sean D. Meehan:** Formal analysis, Investigation, Data curation, Writing – original draft, Writing – review & editing. **Mengming Hu:** Investigation, Writing – review & editing. **Matthew B. Veldman:** Conceptualization, Methodology, Supervision, Investigation, Writing – review & editing, Resources. **Sanjoy K. Bhattacharya:** Conceptualization, Methodology, Supervision, Investigation, Writing – review & editing, Resources.

## Declaration of Competing Interest

The authors declare that they have no known competing financial interests or personal relationships influencing the work reported in this paper.

## Data Availability

Zebrafish Optic Nerve Regeneration Metabolomics - 3 Days Post Crush (Original data) (Metabolomics Workbench).

## Appendix Supplementary materials

Image, application 1
